# Hoffmann syndrome: a case report

**DOI:** 10.1186/1755-7682-7-2

**Published:** 2014-01-06

**Authors:** Hemal MS Senanayake, Anujaya D Dedigama, Randil P De Alwis, Kanapathipillai Thirumavalavan

**Affiliations:** 1 Department of Medicine (ward 13), Teaching hospital, Colombo, North, Ragama, Sri Lanka

## Abstract

Hoffmann syndrome is a rare form of hypothyroid myopathy in adults characterized by presence of muscle weakness, stiffness and pseudohypertrophy. We report a 39 year old male with primary hypothyroidism diagnosed at childhood and not on regular thyroxine therapy who presented with fatigue, cold intolerance, constipation, exertional breathlessness, progressive proximal muscle weakness and swelling of the legs for one year. Examination revealed pseudohypertrophy of calf muscles with marked symmetrical proximal upper and lower limb weakness. His TSH and Creatine phosphokinase (CPK) levels were significantly elevated and electromyography (EMG) was compatible with myopathic disorder. After institution of thyroxine therapy his weakness improved markedly and pseudohypertrophy regressed in two months. We report this case because of its rarity.

## Introduction

Myopathic changes are observed in 30–80% of patients with hypothyroidism [1]. Patients with more severe or longstanding untreated hypothyroidism are more likely to develop clinically significant muscle disease. There are four variants of hypothyroid myopathy known as Hoffmann syndrome, Kocher-Debre-Semelaigne syndrome, atrophic form and myasthenic syndrome [2,3]. Hoffmann syndrome is a specific and very rare form of hypothyroid myopathy which is characterized by proximal weakness, muscle stiffness and pseudohypertrophy in adults [4]. It was first described by Johann Hoffmann in 1897 [5]. Muscle stiffness and pseudohypertrophy is seen in less than 10% hypothyroid patients [1,6].

### Case report

**Figure 1:**
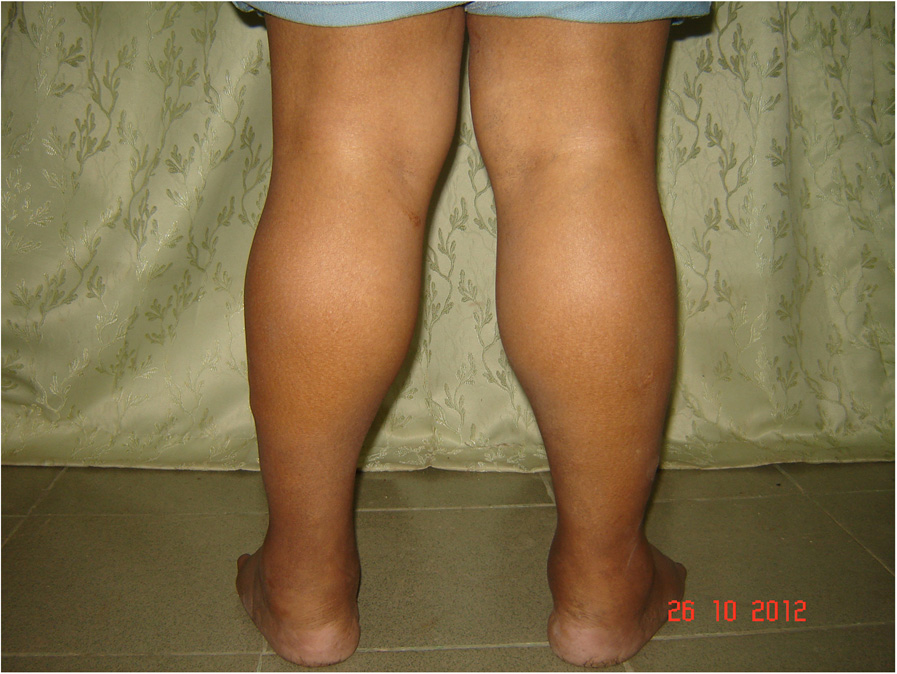
Pseudoypertrophy of calf muscles.

A 39 year old male presented with progressive swelling of the body and constipation for one week. His mother gives a history of poor height gain and mental retardation since the age of 7 years. He was subsequently diagnosed to have primary hypothyroidism. But his treatment compliance was very poor. On examination he was pale. His height was 151 Centimeters and weight was 83 Kilograms with a BMI of 35. His voice was hoarse and had a coarse skin. Macroglossia was observed. There was no goiter. His calf muscles were hypertrophied bilaterally with non-pitting oedema over the ankles (Figure 1). On neurological examination he was mentally retarded. He had proximal upper and lower limb muscle weakness (power 4/5) and generalized hyporeflexia. Cardiovascular examination showed sinus bradycardia (58 beats /minute) with blood pressure of 130/80 mmHg.

Laboratory studies revealed of him having hemoglobin of 10.5 mg/dl, elevated serum cholesterol (295 mg/dl), elevated Thyroid stimulation hormone level (TSH) 25.2 mIU/ml (0.4-4.0) and decreased levels of thyroxin (T_4_) 1.73 μg/dl ( 4–12) and plasma Triido thyronine (T_3_) 19.8 ng/dl (60–120). Furthermore he had elevated Aspartate Aminotransferase (AST) 85 U/L (0–35 U/L), Creatine Phosphokinase (CPK) 959 U/L (85–170), Lactate Dehydrogenase (LDH) 660 U/L (225–450) and serum Aldolase 7.6 U/L (0–7). Sensory and motor conduction studies were normal. Electromyography (EMG) of medial Gastrocnemius, Biceps Brachii, Rectus Femoris and Paraspinal muscles revealed low amplitude and short duration motor unit action potentials (MUAPs) with early recruitment suggestive of a myopathic disorder. The patient was diagnosed of having severe hypothyroidism with Hoffman syndrome.

With these findings patient was started on levothyroxine 125 μg and Atorvastatin 20 mg daily. After two months therapy his hypothyroid symptoms improved, weight came down to 72 Kg, proximal limb weakness disappeared and pseudohypertrophy of the calf muscles regressed.

## Discussion

Hoffman syndrome is a very rare form of hypothyroid myopathy seen in adults with longstanding untreated hypothyroidism. It is characterized by proximal limb muscle weakness and muscle (pseudohypertrophy) [4]. Patients present with muscle cramps, muscle stiffness, weakness, hyporeflexia and delayed deep tendon reflexes [2]. Muscle enlargement (pseudohypertrophy) is very rare and its etiology remains controversial [6]. Calf muscles (gastrocnemius) are almost always involved [1]. The thigh, arm and forearm muscles are involved to a lesser extent [1]. Postulated mechanisms for muscle pseudohypertrophy includes increase deposition of Glycosaminoglycans, increase muscle fiber size and number [1,4,6]. It is associated with change in muscle fiber type from fast twitch type II to slow twitch type I and alteration of oxidative muscle enzyme activity with decreased calcium ATPase activity of fast twitch type II fibers leading to delayed relaxation [1,4]. Mild to moderate elevation of serum CPK level is seen in 70-90% patients with hypothyroidism indicative of muscle involvement but does not correlate with the severity of weakness [4,5]. Other enzymes such as AST, LDH and Aldolase may also be elevated as seen in our patient [2]. Electrophysiological studies may show myogenic, neurogenic or mixed patterns in hypothyroid myopathy [2]. The EMG of myogenic pattern shows reduce duration and amplitude of MUAPs with early recruitment of short action motor units, spontaneous fibrillations and complex repetitive discharges on voluntary muscle contraction [2]. Biopsy of the affected muscles may show muscle fiber necrosis, atrophy, hypertrophy with increased number of nuclei and increased connective tissue [2,5].

Hoffmann syndrome carries favorable prognosis once hormone replacement is instituted and most of the symptoms regress slowly with time, including the muscle enlargement as was the case in our patient. The decline of the muscle enzyme levels occurs slowly, varying from weeks, months or even years with the treatment [2,3]. Electrophysiological findings may persist in some patients despite improvement of symptomatology and muscle enzymes [2].

## Conclusion

Hoffmann syndrome should be considered with other differential diagnosis (Becker’s, Duchenne’s muscular dystrophy, Amyloidosis and focal myositis) when a patient with calf muscle hypertrophy is evaluated and myopathic disorder is suspected, since it is treatable and mostly reversible.

### Consent

Written informed consent was obtained from the guardian of this patient for publication of this case report and accompanying images. A copy of the written consent is available for review by the Editor-in-Chief of this journal.

## Abbreviations

TSH: Thyroid stimulating hormone; CPK: Creatine phosphokinase; EMG: Electromyography; BMI: Body mass index; AST: Aspartate amino transferase; LDH: Lactate dehydrogenase; MUAPs: Motor unit action potentials.

## Competing interests

The authors declare that they have no competing interests.

## Authors’ contributions

HMSS drafted the manuscript. ADD and RPDA involved with literature survey. KT did the critical revision for important intellectual content in the manuscript and given the final approval of the version to be published. All the authors read and approved the final manuscript.
